# High-Grade Serous Carcinoma of the Ovary: A Case Report

**DOI:** 10.7759/cureus.110751

**Published:** 2026-06-12

**Authors:** Farah Jiandani, Savita Somalwar, Anuja Bhalerao

**Affiliations:** 1 Department of Obstetrics and Gynaecology, N.K.P. Salve Institute of Medical Sciences and Research Centre, Nagpur, IND

**Keywords:** high-grade serous ovarian carcinoma, international federation of gynaecology and obstetrics (figo) staging, post-menopausal, staging laparotomy, tumor-node-metastasis (tnm) staging

## Abstract

Ovarian cancer (OC) is one of the most common gynecological cancers, representing the abnormal and uncontrolled growth of ovarian cells leading to the formation of tumors. It is one of the leading causes of death in the majority of nations*.* A wide range of factors, such as lifestyle, environmental factors, genetic factors, hormonal changes, and repeated miscarriages, influence the emergence of OC. Contrary to these factors, breastfeeding, pregnancy, oral contraceptive pills (OCP), and tubal ligation lower the risk of getting OC. The classification of OC as early-stage, advanced-stage, low-grade, or high-grade cancer mainly depends on the histological subtype present at the time of detection. There are various classification systems used to classify OC, but the two most commonly used ones are the International Federation of Gynaecology and Obstetrics (FIGO) staging system and the tumor-node-metastasis (TNM) staging system. The present case is of a 63-year-old female patient diagnosed with high-grade serous carcinoma of the ovary with a TNM staging grade of T_1a _N_0 _M_0 _and an FIGO A1 grade, managed effectively with staging laparotomy. The case study concludes that early identification and care of OC can prevent disease progression, resulting in a better quality of life and prolonging the patient's life. It is crucial to conduct frequent evaluations of a specific age group in the older population to avoid such occurrences.

## Introduction

The leading cause of death worldwide and the barrier to obtaining acceptable longevity in the majority of nations is cancer. The third most frequent gynecological cancer, following uterine and cervical cancer, is ovarian cancer (OC). In this type of cancer, certain ovarian cells become abnormal, and uncontrolled growth leads to a tumor. Additionally, it has the greatest fatality rate and the poorest life expectancy [1].

Ovarian cancer is often categorized as either early- or advanced-stage, low- or high-grade malignancy, but this primarily depends on the histological subtype at the time of its first identification. The International Federation of Gynaecology and Obstetrics (FIGO) staging method and the tumor-node-metastasis (TNM) staging method are the two approaches used to stage OC [2]. A previous study described that staging laparotomy assisted in improving the treatment quality of patients with early-stage OC. The patient diagnosed with stage IA or IB, grade 1 OC by surgical staging laparotomy can have a better prognosis without chemotherapy [3]. Considering the increasing incidence of OC and its effects on a woman's quality of life, it is crucial to come up with a strategy to quickly diagnose the disease and successfully manage it by reducing the possibility of worsening the condition.

## Case presentation

Case description

A 63-year-old female, married for 45 years and post-menopausal for 17 years, reported to the outpatient department of Obstetrics and Gynaecology with chief complaints of abdominal pain for 15 days and a lump with swelling in the lower abdomen that had gradually increased in size over 7 months. The patient's obstetric history revealed a parity of 7, with 3 living children and 5 deaths (P7L3D5). Of the five deaths, two deaths were due to twin pregnancy, one death was at the age of 4 years of the child, and one death was at the age of 3 weeks of the neonate. The patient also reported a history of blood transfusion 20 years back with 20 units of packed red blood cells.

Physical examination

On physical examination, the patient was vitally stable, with signs of pallor. On per-abdominal examination, the abdomen was tender on the left side, and a single abdominal lump of size 25 cm × 20 cm was palpated in the umbilical, hypogastric, lumbar, and iliac regions. The lump was firm in consistency, smooth and mobile in the horizontal plane with ill-defined margins and a non-palpable lower border. An extension of a lump in the pelvis was also observed, which suggested the possibility of ovarian involvement. Per vaginal examination showed an atrophied cervix with a mass of a gravid uterus of 26 weeks in size.

Diagnostic assessment

For further investigations, a diagnostic assessment consisting of a Papanicolaou smear test (PAP) and ultrasonography (USG) was performed to determine the cause. The result of the PAP smear was suggestive of an inflammatory low-grade squamous intraepithelial lesion, and USG of the abdomen and pelvis revealed an atrophic uterus of 5.6 cm × 2.5 cm × 2.1 cm and a large, ill-defined heterogeneous mass of size 16.3 cm × 9.5 cm × 4.7 cm that was hypoechoic, solid, and located anterosuperiorly to the fundus of the uterus. The mass was seen to be epi-centered in the pelvis and extending up to the umbilical region. Furthermore, contrast-enhanced computed tomography (CECT) was performed to confirm the above diagnosis, which demonstrated a large, ill-defined, heterogeneous soft tissue attenuation lesion (Hounsfield Units (HU) +13 to +69), likely arising from the right ovary, measuring 11.0 cm × 13.8 cm × 16.8 cm, with a few calcific foci (Figure 1).

**Figure 1:**
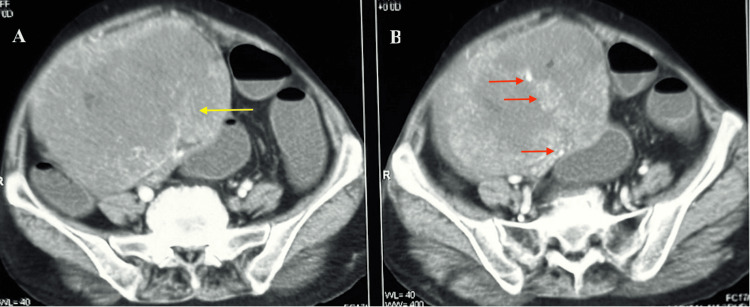
Contrast-enhanced computed tomography (CECT) of high grade serous carcinoma of the ovary The yellow arrow in Panel A points to a large, ill-defined, heterogeneous soft tissue attenuation lesion measuring 11.0 cm × 13.8 cm × 16.8 cm. The calcific foci are marked with red arrows in Panel B.

The right ovary was not observed separately from the lesion. On post-contrast study, the lesion showed significant heterogeneous enhancement with a few non-enhancing areas within. The lesion appeared to be highly vascular with multiple tortuous collaterals supplying and draining this lesion. Mild free fluid was also observed surrounding the lesion. Based on the above investigations, the possibility of right ovarian malignant etiology may be ruled out.

The patient was advised to undergo an exploratory staging laparotomy, for which written informed consent was obtained before commencement of the procedure. The procedure for staging laparotomy was performed under all aseptic precautions under general anesthesia with the patient in a supine position. Betadine painting and draping were done. A midline vertical incision was made from the pubic symphysis up to the xiphisternum, and the abdominal layers were opened step by step till the appearance of the peritoneum. The mass of 16 x18 x 20 cm in size, which was highly vascular and firm in consistency, was observed in the right ovary (Figure 2).

**Figure 2:**
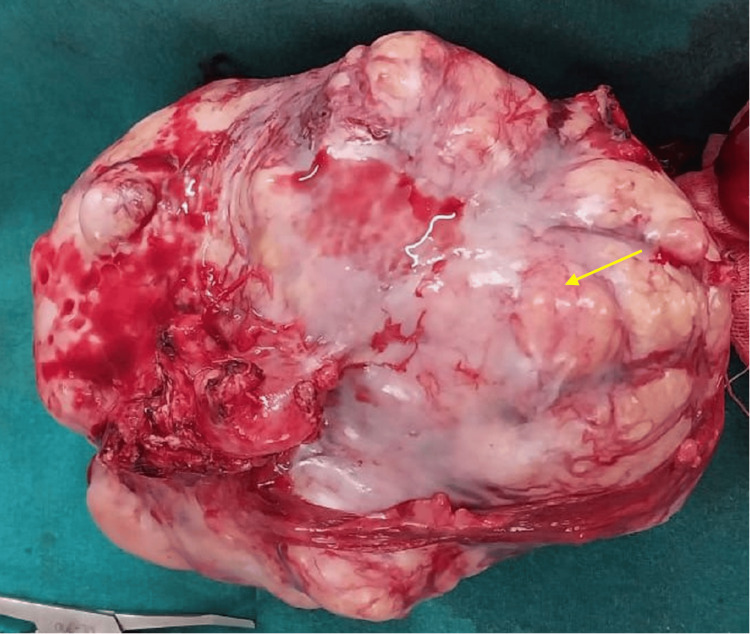
Specimen illustrating high-grade serous carcinoma of the ovary The yellow arrow indicates a highly vascular and firm mass of 16 x 18 x 20 cm in size.

Dissection and devascularization of the right fallopian tube were done using the clamp, cut, and ligate technique. The mass was removed from the right ovary and sent for histopathological examination (HPE) and cytopathological examination along with the right ovary and the right fallopian tube. After achieving hemostasis, an abdominal wash was given. The abdomen and skin were sutured back in layers along with the insertion and fixation of an abdominal drain. The procedure was uneventful, and the patient adhered well to the treatment.

The HPE confirmed the malignant nature of the disease, which was high-grade serous carcinoma of the right ovary with a TNM staging grade of T_1a_ N_0_ M_0_ and an FIGO A1 grade, which represents that only one ovary was affected by the malignancy, the ovarian surface was free of tumor, and the capsule was intact (Figure 3). The cytopathology report was suggestive of epithelial malignancy, along with positive results for the presence of peritoneal fluid. On postoperative day 3, no gap, discharge, or mis-approximation was observed at the site of incision.

**Figure 3:**
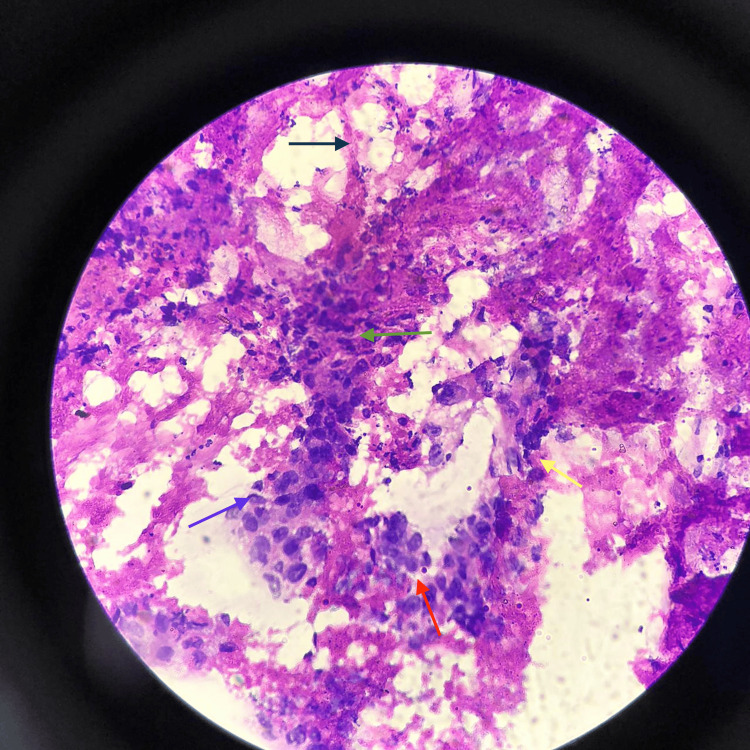
Histopathological examination (HPE) confirmed the high-grade serous carcinoma The black arrow points at tumour necrosis with scattered malignant cells; the green arrow marks the high-grade atypical epithelial cluster (solid sheets), indicating loss of architecture; the yellow arrow points at the tumour cells in a necrotic background; the red arrow points at the enlarged nuclei; and the blue arrow points at the marked nuclear pleomorphism and hyperchromasia.

Therapeutic intervention

The patient was given one unit of packed red blood cells each pre-operatively, intra-operatively, and post-operatively. The patient was referred to a cancer hospital for chemotherapy, which consisted of tablet domeperidone** **10 mg thrice a day for five days; milk of magnesiathree spoons and pregabalin 75 mg before going to bed, for five days; and providone iodine** **gargles six to eight times a day. The cycle was repeated every week for nine doses. Additionally, the medications prescribed involved injection (Inj) ceftriaxone** **1 gm intravenous (IV) twice a day (BD) for 12 days and then continued 200 mg orally BD for 5 days along with Inj metronidazole** **100 cc IV BD for 9 days and continued 400 mg thrice a day (TDS) for 5 days and levofloxacin** **500 mg BD for 2 days, which was an antibiotic used against bacterial infection, tablet Enzomac (bromelain 90 mg + trypsin 48 mg + rutoside 100 mg; Macleods Pharmaceuticals, Mumbai, India) TDS and tablet Combiflam (ibuprofen 400 mg + paracetamol 325 mg; ​​​​​​Sanofi Consumer Healthcare India, Mumbai, India)** **TDS for 10 days to treat pain and inflammation. Adequate fluid intake and an iron and protein-rich diet were prescribed for further management of the condition. The patient was advised to follow up in the Gynaecology department after 2 weeks and to inform immediately in case of abdominal pain, increased per-vaginal bleeding, or foul-smelling discharge.

## Discussion

The present case is of a 63-year-old female patient who described early symptoms of pain and swelling in the lower abdominal region and was diagnosed with high-grade serous carcinoma of the ovary with TNM staging grade of T_1a_ N_0_ M_0_ and a FIGO A1 grade. As the case was in the initial stage of OC, it was managed with a surgical approach that consisted of staging laparotomy followed by chemotherapy sessions. The primary objectives of the case were early detection, appropriate management of OC in its initial phase, and prevention of complications associated with it.

The results of a previous study demonstrated that a variety of variables, including lifestyle, environmental, and genetic factors, hormonal changes, and multiple miscarriages, influence the development of OC. In contrast, oral contraceptive pills (OCP), breastfeeding, and pregnancy reduce the chances of developing OC [1]. Additionally, patients who have not undergone the process of tubal ligation have a high chance of developing OC [4-6]. In the present case, the patient described a history of multiple miscarriages, which possibly contributed to the development of OC in her later life.

Previous research has revealed that older women are more prone than younger women to have an advanced stage of OC at the time of detection or due to delayed diagnosis of the condition [2]. Even though the prevalence of early diagnosis is low, thorough assessment in such conditions helps manage the progression of the condition over a specific period of time. To reduce such incidences, it is essential to perform routine evaluation after a certain interval for the age group in the elderly population [7].

Women who experience abdominal bloating, early satiety, decreased intake of food, pain in the abdomen or pelvis, elevated urine urgency or frequency, unjustified loss of weight, tiredness, or altered bowel habits should be frequently evaluated in a primary care setting and can then be transferred to secondary care to obtain a quicker diagnosis and therapeutic intervention, consequently increasing the survival rate. In a previous study, the majority of women referred were premenopausal, where the risk of OC is minimal. If the patient is having previously mentioned complaints for a prolonged period of time, the preferred investigation method is USG, which shows an ill-defined heterogeneous mass in case of a tumor, based on which further investigation is required for confirmation of the diagnosis [8].

Combining surgery with chemotherapy, which should be platinum-based, is considered the cornerstone of treating OC. Women with cancer in its initial stages and those with advanced disease had the most favourable prognosis after complete removal of the tumor mass [8]. A study also suggested that patients who underwent surgical staging laparotomy for the treatment of stage IA or IB, grade 1 OC have a favourable prognosis without undergoing chemotherapy [3]. Women with stage I epithelial OC get the same outcome following laparoscopic and open surgery. Laparoscopic treatment in the early stages of OC is safe and effective for those with stage I epithelial carcinoma [9,10]. There is currently insufficient proof to support either the equality or superiority of laparoscopic or robotic staging of early epithelial OC over laparotomy [11-13]. Hence, in the present case, a staging laparotomy was performed as part of the surgical intervention for the removal of the tumor.

## Conclusions

The present case report demonstrated a case of an elderly woman with an ill-defined heterogeneous mass arising from the right ovary, which on diagnosis confirmed high-grade serous carcinoma of the ovary with TNM staging grade of T_1a_ N_0_ M_0_ and a FIGO A1 grade. The ovarian carcinoma was effectively managed with staging laparotomy, along with the use of antibiotics. Hence, in conclusion, the case report illustrates that early detection and management of OC will prevent further progression of the disease, leading to improved quality of life and consequently enhancing the life span of the patient.

## References

[1] Momenimovahed Z, Tiznobaik A, Taheri S, Salehiniya H (2019). Ovarian cancer in the world: epidemiology and risk factors. Int J Womens Health.

[2] Main C, Chen X, Zhao M, Chamley LW, Chen Q (2022). Understanding how pregnancy protects against ovarian and endometrial cancer development: fetal antigens may be involved. Endocrinology.

[3] Tabata T (2019). Staging laparotomy in early ovarian cancer. Surgery for Gynecologic Cancer. Comprehensive Gynecology and Obstetrics.

[4] van Bommel MH, IntHout J, Veldmate G, Kets CM, de Hullu JA, van Altena AM, Harmsen MG (2023). Contraceptives and cancer risks in BRCA1/2 pathogenic variant carriers: a systematic review and meta-analysis. Hum Reprod Update.

[5] Matulonis UA, Sood AK, Fallowfield L, Howitt BE, Sehouli J, Karlan BY (2016). Ovarian cancer. Nat Rev Dis Primers.

[6] Cibula D, Widschwendter M, Májek O, Dusek L (2011). Tubal ligation and the risk of ovarian cancer: review and meta-analysis. Hum Reprod Update.

[7] Zhao D, Zhou Z, Shen C (2022). The effect of health check-ups on health among the elderly in China: evidence from 2011-2018 longitudinal data. Int J Public Health.

[8] Sundar S, Neal RD, Kehoe S (2015). Diagnosis of ovarian cancer. BMJ.

[9] Ran X, He X, Li Z (2022). Comparison of laparoscopic and open surgery for women with early-stage epithelial ovarian cancer. Front Oncol.

[10] Kong Q, Wei H, Zhang J, Li Y, Wang Y (2021). Comparison of the survival outcomes of laparoscopy versus laparotomy in treatment of early-stage ovarian cancer: a systematic review and meta-analysis. J Ovarian Res.

[11] Park HJ, Kim DW, Yim GW, Nam EJ, Kim S, Kim YT (2013). Staging laparoscopy for the management of early-stage ovarian cancer: a metaanalysis. Am J Obstet Gynecol.

[12] Rutten MJ, Leeflang MM, Kenter GG, Mol BW, Buist M (2014). Laparoscopy for diagnosing resectability of disease in patients with advanced ovarian cancer. Cochrane Database Syst Rev.

[13] Finch L, Chi DS (2023). An overview of the current debate between using minimally invasive surgery versus laparotomy for interval cytoreductive surgery in epithelial ovarian cancer. J Gynecol Oncol.

